# A survey of ontology learning techniques and applications

**DOI:** 10.1093/database/bay101

**Published:** 2018-10-05

**Authors:** Muhammad Nabeel Asim, Muhammad Wasim, Muhammad Usman Ghani Khan, Waqar Mahmood, Hafiza Mahnoor Abbasi

**Affiliations:** 1 Al-Khawarizmi Institute of Computer Science (KICS), University of Engineering and Technology, Lahore, Pakistan; 2 Department of Computer Science and Engineering, University of Engineering and Technology, Lahore, Pakistan

## Abstract

Ontologies have gained a lot of popularity and recognition in the semantic web because of their extensive use in Internet-based applications. Ontologies are often considered a fine source of semantics and interoperability in all artificially smart systems. Exponential increase in unstructured data on the web has made automated acquisition of ontology from unstructured text a most prominent research area. Several methodologies exploiting numerous techniques of various fields (machine learning, text mining, knowledge representation and reasoning, information retrieval and natural language processing) are being proposed to bring some level of automation in the process of ontology acquisition from unstructured text. This paper describes the process of ontology learning and further classification of ontology learning techniques into three classes (linguistics, statistical and logical) and discusses many algorithms under each category. This paper also explores ontology evaluation techniques by highlighting their pros and cons. Moreover, it describes the scope and use of ontology learning in several industries. Finally, the paper discusses challenges of ontology learning along with their corresponding future directions.

## Introduction

At start of the 21st century, with the advancement of technologies in different domains, unstructured data on the internet in the form of electronic news and scientific literature grew exponentially. However, the web at start of the 21st century was not efficient. If one author wrote about some topic on one website, another author could provide contradictory information about the same topic on another website. In other words, the web was disconnected, inconsistent and dumb. Extracting useful information from such type of web was an erroneous process. In order to tackle this problem, the concept of the semantic web was introduced by Maedche and Staab in 2001 (1). The underlying motivation behind this idea was to create a web platform that should be highly linked, consistent and intelligent. Ontologies play a fundamental role to implement the idea of the semantic web.

An ontology is a formal and structural way of representing the concepts and relations of a shared conceptualization (2). More precisely, it can be defined as concepts, relations, attributes and hierarchies present in the domain. Ontologies can be created by extracting relevant instances of information from text using a process called ontology population. However, handcrafting such big ontologies is a difficult task, and it is impossible to build ontologies for all available domains (3). Therefore, instead of handcrafting ontologies, research trend is now shifting toward automatic ontology learning.

Whenever an author writes something in the form of text, he is actually doing it by following a domain model in his mind. He knows the meanings behind various concepts of particular domain, and then using that model, he transfers some of that domain information in text, both implicitly and explicitly.

**Figure 1:**
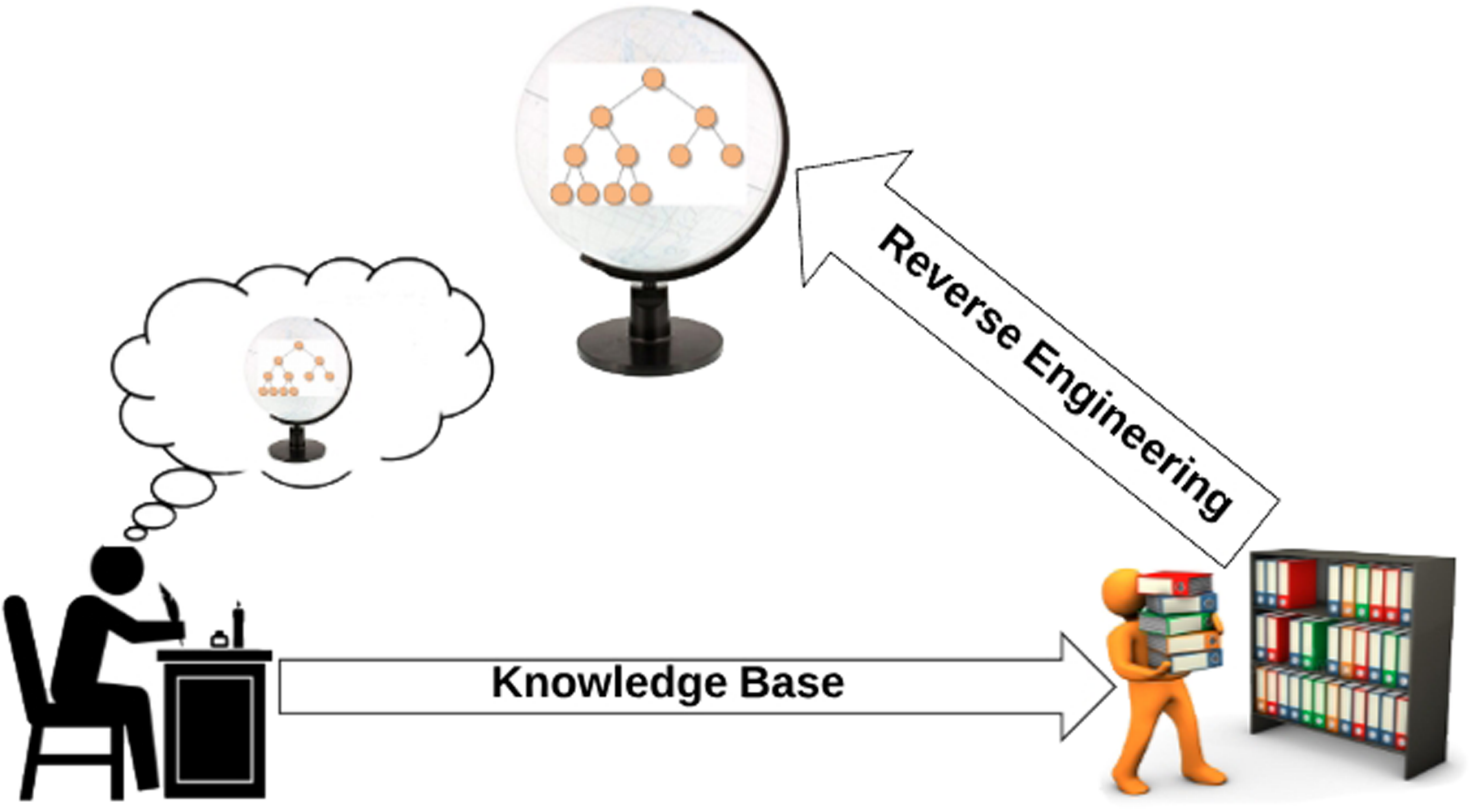
Ontology learning from text: reverse engineering task.

Ontology learning is a reverse process as domain model is reconstructed from input text by exploiting the formal structure saved in author’s mind (4). The entire reconstruction process of domain model is illustrated in Figure 1.

**Figure 2:**
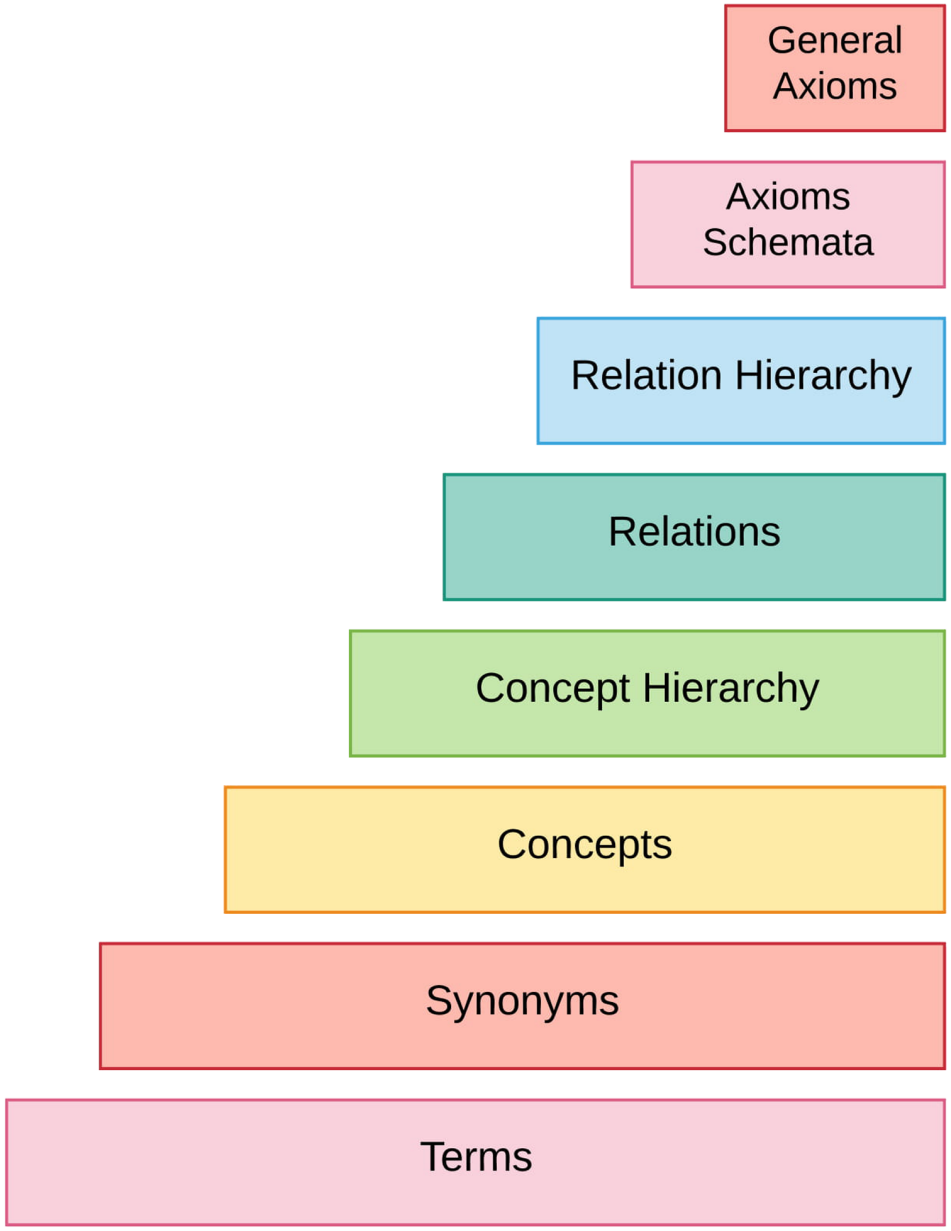
Ontology learning layer cake.

Figure 2 summarizes different steps required to accomplish an ontology from unstructured text.

The process of ontology acquisition starts by extracting terms and their synonyms from underlying text. Then corresponding terms and synonyms are combined to form concepts. After that, taxonomic and non-taxonomic relations between these concepts are found. Finally, axiom schemata are instantiated and general axioms are extracted from unstructured text. This whole process is known as ontology learning layer cake.

## Summary of previous surveys

This section summarizes previous work done in domain of ontology learning and highlights their contributions along with found research gaps. Ding and Foo (5) published a survey in 2002 that summarized characteristics of 12 ontology learning systems. They provided details about various ontology learning algorithms and highlighted different problems that were encountered by these systems during ontology learning. Findings of this survey are summarized as follows: (i) most of the ontology learning systems were learning the ontology by the help either of seed words or base ontology instead of building it from scratch, (ii) natural language processing techniques for concept extraction had revealed promising results, whereas (iii) relation extraction was one of the major challenges in natural language processing and it caused hindrance for ontology learning systems.

In 2003, OntoWeb Consortium (6) published a report on 36 most relevant methodologies and tools used for the sake of ontology learning from unstructured text. Key points of this survey are discussed below: (i) the survey discussed 36 ontology learning systems but lacked proper classification hierarchy and (ii) most of the systems discussed in this survey were semi-automated and it lacked research exposure toward automated ontology learning systems.

Shamsfard and Barforoush (14) published another survey report around the same time. They classified and compared various ontology learning systems on the basis of following three categories: (i) starting point of ontology learning system (i.e. either ontology is built from scratch or it uses some pre built base ontology), (ii) what kind of ontology was needed by the application (e.g. a scientific application may need short, axiomatized ontology to solve its problems) and (iii) degree of automation. In their survey, seven prominent ontology learning systems namely ASIUM (7), Doodle II (8), Hasti (9), Svetlan (10), Syndikate (11), Text-to-Onto (12) and WebKB (13) were analyzed. Critical analysis of this survey leads to following conclusions: (i) this survey highlighted research on extraction of taxonomic relations but did not explore non-taxonomic relations extraction, (ii) most of the explored ontology learning systems needed prior domain knowledge in form of base ontology to extract ontologies from unstructured text and (iii) the authors did not mention any automatic ontology learning system.

In 2005, Buitelaar *et al.* (15) presented a survey of selected papers from two ontology learning workshops. They summarized the contents of ontology learning papers in perspective of methodologies used for ontology extraction, evaluation methods and challenges of various real life application scenarios. They introduced the phrase of ‘ontology learning layer cake’.

In 2007, Zhou (16) published a survey that illustrated the process of ontology learning in detail and highlighted a comprehensive review of open issues and challenges in ontology learning. They proposed a hypothetical model for the development of ontology learning process. Concluding facts of the paper are as follows: (i) they suggested an improvement in those ontology learning systems that did not involve users at any level of ontology learning, (ii) they highlighted the importance of knowledge representation in ontology learning domain and (iii) need to move from coarse relationship classes to fine relationships was elucidated. After critical analysis, we found that this survey overlooked significant logic-based techniques that are used to form axioms.

In 2011, Hazman *et al.* (17) published a survey of various ontology learning approaches. They divided ontology learning into two categories, i.e. learning from unstructured and semi-structured data. One of the key findings in their survey is that natural language processing techniques are considered efficient to learn ontology from unstructured data. Whereas, data mining and web content mining techniques are more applicable when it comes to learning ontology from semi-structured data. In their survey, they discussed ontology learning by using domain keywords but ontology building from scratch was not explored. This survey also highlighted the need and importance of ontology evaluation. They described five levels of ontology evaluation, namely, lexical (vocabulary), hierarchical, contextual, syntactic and structural levels. They concluded that human-based evaluation is possible at all above-mentioned five levels (17).

Our survey paper differs from existing work in various ways, some of which are highlighted below: (i) Previous surveys are outdated and focus on old techniques for ontology learning. This survey considers the latest trends in different tasks of ontology learning layer cake. (ii) Ontology learning techniques are categorized into three classes and explored them at each level of layer cake shown in Figure 2. (iii) This paper thoroughly dives into the industries where ontology learning is being used extensively and highlights the prominent work to motivate researchers in domain of ontology learning. (iv) State-of-the-art ontology learning data sets are also discussed; (v) this survey also extensively discusses various evaluation techniques for ontology learning along with their pros and cons and (vi) it not only highlights the challenges but also suggests possible ways to tackle these challenges. We found 200 research papers using Google Scholar by feeding queries of following words ‘ontology learning, ontology learning evaluation, industrial applications of ontology learning, knowledge extraction, ontology learning algorithms, ontology learning from text, ontology learning from unstructured text, ontology learning methods’ with different combinations. After critically analyzing the retrieved articles, we found 140 research papers as most significant in context of ontology learning, which are discussed in this survey.

## Ontology learning techniques

**Figure 3:**
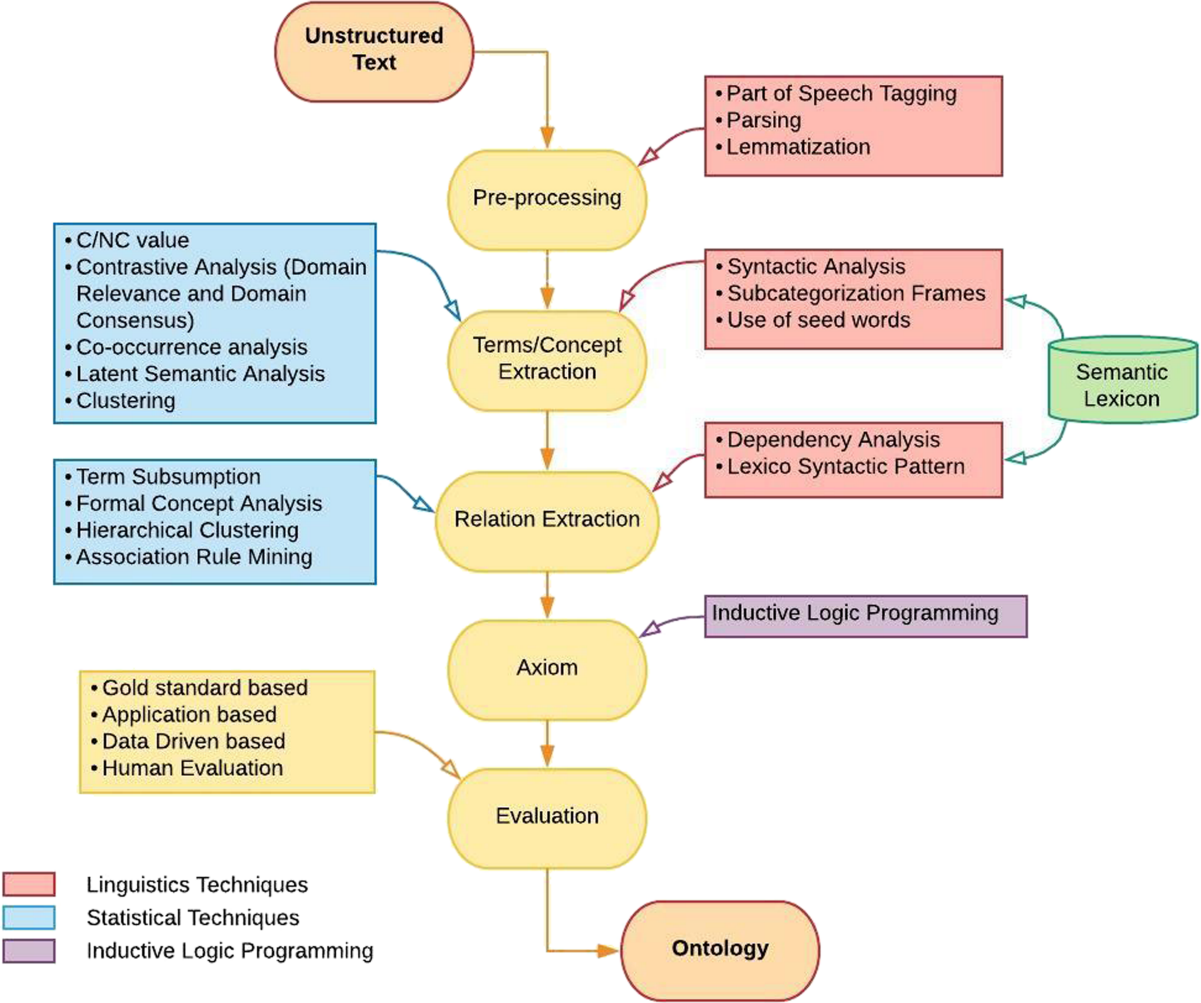
Methodology of ontology learning.

Over the past decade, various techniques from the fields of natural language processing, machine learning, information retrieval, data mining and knowledge representation have contributed for the improvement of ontology development. Data mining, machine learning and information retrieval provide statistical techniques for extracting domain specific terms, concepts and associations among them. On the other hand, natural language processing plays its role in almost every level of ontology learning layer cake by providing linguistic techniques. Formal representation of a developed ontology requires inductive logic programming (ILP) techniques which provide logic simplification and formal representation algorithms. Although ontology learning techniques can be categorized at several levels, we categorize these techniques into three classes namely linguistic, statistical and logical. Figure 3 elucidates various algorithms that fall under three main categories, i.e. linguistic-, statistical- and logical-based, and are used at different levels of ontology learning layer cake.

For the sake of better visualization of classified techniques, Figure 3 is constructed using three different colors red, blue and purple. Red color is used to represent ontology learning techniques that fall into linguistic based class. Similarly, blue and purple colors are used for the representation of algorithms that belong to statistical and logical class, respectively. Flow of algorithms in Figure 3 shows that ontology learning is a step-by-step process. Firstly, text corpora are preprocessed by using linguistic techniques such as part of speech tagging, parsing and lemmatization. After preprocessing, relevant terms and concepts of domain are extracted. This stage utilizes various techniques of natural language processing such as syntactic parsing, subcategorization frames and seed words extraction, along with some techniques from statistical domain like C/NC value, contrastive analysis, co-occurrence analysis, latent semantic analysis (LSA) and clustering. Besides obtaining concept clusters, taxonomic and non-taxonomic relations among these concepts are also required. For this purpose, an amalgam of Natural Laguange Processing (NLP) techniques and statistical approaches are used which includes dependency analysis, lexico-syntactic analysis, term subsumption, formal concept analysis (FCA), hierarchical clustering and association rule mining (ARM). It is also worth mentioning that semantic lexicons are used at both term/concept extraction and relationship extraction stage. In next step, axioms are formed using ILP. To evaluate the integrity of developed ontology, different evaluation measures exist. This paper reviews four ontology evaluation techniques (gold standard-based, application-based, data-driven and human-based) along with their merits and demerits.

### Linguistic techniques

Linguistic techniques are based on characteristics of language and play a key role at almost every stage of ontology learning layer cake. Linguistic techniques are mostly used for preprocessing of data as well as in some other ontology learning tasks such as term, concept and relation extraction. This section first discusses three linguistic techniques for preprocessing namely part of speech tagging, parsing and lemmatization. Secondly, it discusses linguistic techniques used at the stages of terms, concepts and relations extraction in ontology learning process. For terms and concepts extraction, three algorithms, syntactic analysis, subcategorization framing and use of seed words, are discussed; whereas for relationship extraction, dependency analysis and lexico-syntactic patterns are discussed. Details of these algorithms are presented in sections below.

#### Linguistics for pre-processing

This section discusses part of speech tagging, sentence parsing and lemmatization which are linguistic-based preprocessing techniques used in almost every ontology learning methodology.

Part of speech tagging is the process of labeling corpus words to their corresponding part of speech tags. Brill Tagger (18) and TreeTagger (19) are widely used for part of speech tagging because of their better performance. Parsing is a type of syntactic analysis that finds various dependencies between words in a sentence and represents them in the form of a data structure called parsing tree. For sentence parsing, commonly used tools are Principar (20), Minipar (21) and Link Grammar Parser (22). Some parsers are built on statistical parsing systems such as Stanford Parser, which is a lexicalized probabilistic parser (23). Petit *et al.* (24) used Stanford CoreNLP API for part of speech tagging. On the other hand, Drymonas (26) used GATE (General Architecture for Text Engineering, https://gate.ac.uk/) (25) and OpenNLP (https://opennlp.apache.org/) to preprocess the corpus for ontology learning. They claimed that the accuracy of ontology learning was improved with the use of Openly based Pops tagger and parser. In 2001, two unique techniques of Pops tagging [using WordNet (https://wordnet.princeton.edu/)] and parsing (using augmented grammar) were introduced in the context of ontology learning (27).

Lemmatization is another linguistic based preprocessing technique which is used to bring the terms into their normal form. For example, the lemma of ‘running’ and ‘ran’ should be ‘run’.

It is used to reduce the dimensionality of data. It handles various morphological variants of one term. Petit *et al.* (24) utilized Cornel API (https://stanfordnlp.github.io/CoreNLP/api.html) to lemmatize textual data for ontology learning purpose. On the other hand, Drymonas *et al.* (26) did lemmatization using an external tool of WordNet-based Java Library. They claimed that preprocessing of data is important to fetch domain relevant terms.

Importance of data preprocessing in ontology learning can be analyzed from the research work done by Jiang and Tan (28). They compared the performance of two systems [Text-to-Onto and Text-2-Onto (75, 120, 121, 122)] before and after utilizing parsers. To extract concepts for both systems, initial experimentation was done by utilizing hand-crafted rules based on Pops tagging. On same experimental setup, concepts were extracted by introducing Berkley Parser and Stanford Parser in both systems. Their results showed that before using parser, performances of both systems were 47.2 and 74.4%, which boosted up to 92.8 and 92%, respectively, after applying the above mentioned parsers. In a nut shell it can be concluded that **to get a higher accuracy of ontology learning task, efficient preprocessing of data using good linguistic techniques is a necessity**.

#### Linguistics for knowledge extraction

In ontology learning, linguistic techniques are also used for extraction of terms, concepts and relations. After thoroughly analyzing the literature, it can be concluded that syntactic structure analysis and subcategorization frames are used for term extraction. On the other hand, 11 researchers (38, 40, 149, 46, 47, 15, 41, 42, 43, 44, 45) used dependency analysis and lexicon-syntactic patterns for relation extraction. Besides this, lexicons could also be used for the extraction of concepts and relations. Moreover, extraction of domain-specific terms and concepts has improved by introducing seed words in pipeline of ontology learning.

**Term/concept extraction** To extract terms and concepts using syntactic structures, first corpus is tagged with parts of speech. This information is utilized to extract syntactic structures in sentence such as noun phrases and verb phrases. These structures are employed to find terms by analyzing the words and modifiers present in them. For example, in ontology learning, syntactic structure of noun phrase (NP) can be used to extract potential candidate terms from the corpus. Hippisley *et al.* (29) used syntactic analysis and employed head-modifier principal to identify and extract complex terms in which head of the complex term takes the role of hypernym. For example, the complex term ‘acute appendicitis’ will be extracted as a potential term candidate because the head of these terms, ‘appendicitis’, is taking the hypernym role. On Chinese text, this technique was able to achieve the accuracy of 83.3%.

Subcategorization frame is another concept of linguistic theory that can be employed in ontology learning tasks (30, 31). Subcategorization frame of a word is the number of words of a certain form that it selects when appearing in a sentence. For example, ‘Bob writes a letter’. In this sentence, the verb ‘to write’ chooses ‘Bob’ and ‘letter’ as its neighboring words so its subcategorization frame consists of these two words. In other words, a restriction of selection is now made for the verb ‘write’ that it will select its neighboring words from the classes of ‘Person’ and ‘written-communication’. When used in conjunction with clustering techniques, this restriction of selection is able to discover concepts (32).

Use of seed words is another common methodology that is employed to guide many ontology learning tasks (33). Seed words are domain-specific words that provide a base for other algorithms to extract similar domain specific terms and concepts (34). This technique ensures that only those terms that are more relevant and semantically closer to seed words are extracted. Sanchez and Moreno *et al.* (35) made use of seed words to extract domain-specific documents from the web and used them as corpus to extract terms and concepts for ontology construction. However, Fraga and Vegetti (36) manually put the seed words in a text file to ease the extraction process.

##### Relation Extraction

Dependency analysis helps in finding relations between terms by using dependency information present in parsing trees (37). Ciaramita *et al.* (38) used dependency paths information present in parse trees to find relationship patterns. For two specific concepts, they found relations by extracting the shortest path among those concepts in parsing tree. Their approach was able to learn 83.3% correct relations from corpus. Besides this, it was also used by Sordo *et al.* (39) as relation extraction technique. Lexico-syntactic pattern is a rule-based approach that plays its role in taxonomic and non-taxonomic relation extraction phases of ontology learning. To extract relations, this algorithm makes use of regular expressions. For example, ‘NP such as NP, NP, … , NP’ is a rule that will extract patterns like ‘seasons, such as summer, winter, autumn, and spring’. This type of rule-based approach is quite helpful in extracting is–a relationship, i.e. is a (summer, season). On the other hand, lexico-syntactic patterns like ‘NP is a part of NP’ can be used to extract non-taxonomic relationships. In 1998, Hearst (40) introduced an algorithm that enabled the extraction of different types of lexico-syntactic patterns. She extracted 106 relations from *New York Times* corpus in which 61 relations were validated by WordNet. In other words she obtained a minimum accuracy of 75.55%. Besides this, Sombatsrisomboon *et al.* (149) used these patterns for extraction of taxonomic relations. Buitelaar (15), Kaushik and Chatterjee (41), Ismail *et al.* (42, 43), Panchenko *et al.* (44) and Atapattu *et al.* (45) also used these patterns in their work and concluded that lexico-syntactic patterns provide a reasonably good precision. However, the manual effort required to produce these patterns from data sets is also very extensive. Therefore, Snow *et al.* (46) made effort in extracting such patterns by using machine learning algorithms. Using logistic regression on a training set of known hypernyms pairs, they automatically learned dependency paths from parse tree and subsequently used them to extract new relationships in unknown data.

Semantic lexicons are knowledge resources in the domain of ontology that play a vital role at different levels of ontology learning (47). Famous semantic lexicons include WordNet (https://wordnet.princeton.edu/) and Unified Medical Language System (https://www.nlm.nih.gov/research/umls/). Semantic lexicons can be used to extract terms, concepts and taxonomic and non-taxonomic relations. They offer a wide range of predefined concepts and relations. These concepts are organized into set of similar words called synsets (sets of synonyms). In (48), Turcato *et al.* used these synsets for the formation of concepts. Besides this, semantic lexicons also have a number of predefined associations like hypernymy, meronymy etc. They have been employed by Navigli *et al.* (49) for extraction of taxonomic and non-taxonomic relations.

### Statistical techniques

Statistical techniques are solely based on statistics of the underlying corpora and do not consider underlying semantics. Most of the statistical techniques make extensive use of probabilities and are frequently used in early levels of ontology learning after linguistics preprocessing. These techniques are mostly used for term extraction, concept extraction and taxonomic relation extraction. Statistical techniques include C/NC value, contrastive analysis, clustering, co-occurrence analysis, term subsumption and ARM. This section briefly discusses these techniques.

#### Term and concept extraction

Ontology learning layer cake starts with the tasks of term extraction and concept extraction. Some of the techniques that are used for these tasks include C/NC value, contrastive analysis and co-occurrence analysis. They are discussed below.

##### C/NC value

C/NC value is used for multi-word terminology extraction. Terminologies are domain specific multi-word terms or a group of terms that can form a valid concept. C/NC value technique receives various multi-word terms as input and returns a score for each of them. This score is a combination of two values, i.e. CValue and NCValue. C value tends to find a group of terms that are valid in the corpus. In other words, it looks for term-hood of the multi-word terms. Whereas, NC value is a modification in C value that considers the context of multi-word term and tries to find longer strings that appear more frequently in the corpus. These longer groups of words can then form the basis of concepts. Mathematically, C value can be calculated as (50)

$$ Cvaluez=\left\{\begin{array}{cc}{\mathit{\log}}_2\left|a\right|.f(a)&\ \textrm{if}\left|a\right|=g\\{}{\mathit{\log}}_2\left|a\right|.f(a)-\frac{1}{C(a)}\sum_{k=1}^{C(a)}\times f\left({b}_k\right)& \textrm{otherwise}\end{array}\right. $$

where

- *g* is maximum term candidate size in number of words,
- *a* is the multi-word term candidate,
- *f (a)* counts the frequency of *a* in the corpus,
- *C(a)* is the number of longer strings that contain term candidates,
- *b_k_* is the longer strings that contain term candidates.

For example, soft contact lens is a candidate term that also has smaller terms like soft contact and contact lens. Contact lens is an independent term and it can appear independently in the corpus; therefore, C value for this term will be high as compared to soft contact. List of words is then ranked according to their C value scores.

Once C value is found, the next step is to incorporate contextual information. Context words in a window of one word (from left and right side of candidate term) are extracted as a list. These words are then assigned a weight using formula below:

(1) \begin{equation*} \mathrm{weight}\left(\mathrm{t}\right)=\frac{\mathrm{f}\left(\mathrm{t}\right)}{\mathrm{n}} \end{equation*}

where t is the context word, f (t) stands for number of multi-word terms that have term t as their context word and n stands for the total number of multi-word terms.

A weight value is then added into C value to get the NC value. Mathematically, it can be written as

(2) \begin{align*} NCValue&=0.8\left( CValue(a)\right)\nonumber\\&+0.2\left({\sum}_{t\ \epsilon C(a)}{f}_a(t)\left( weight(t)\right)\right) \end{align*}

In above equation,

a stands for the candidate term

- C_a_ is the set of context words of *a*
- t is one such candidate term from C_a_
- f_a_(t) is the frequency of the word b as context term of a
- weight(t) is the weight as calculated in Equation 1
- 0.8 and 0.2 are optimized factors provided by Frantzi *et al.* (50).

Frantzi *et al.* (50) introduced C/NC value and claimed that use of C value instead of pure frequency tends to increase the precision of extracted terms. They also concluded that use of contextual information in NC value ensures a higher concentration of real terms at the top of extracted terms list. Drymonas (26) employed C/NC value to extract multi-word concepts from OHSUMED (51) and computer science corpus (52) for ontology learning. They evaluated first 150 extracted terms with the help of domain experts. For computer science corpus, they obtained 86.67% precision and 89.6% recall, whereas for medical corpus, 89.7% precision and 91.4% recall were obtained, which showed effectiveness of this statistical measure. Besides this, in 2016, Yang *et al.* (53) and Chandu *et al.* (54) used C/NC value to develop their automatic question answering framework for BioASQ challenge. It was used to extract candidate concepts from biomedical domain. C/NC value showed promising results in 2016 BioASQ challenge; therefore, researchers continued its use in 2017 challenge as well.

##### Contrastive analysis

Term extraction process can extract terms that are not relevant to the domain of the corpus. These terms need to be filtered out. Contrastive analysis is a technique that filters out terms obtained through term extraction procedure (55). In 2003, Navigli *et al.* (49) introduced two new measures for contrastive analysis technique in the domain of ontology learning, namely domain relevance and domain consensus. They use two types of corpora, relevant corpus (target domain) and non-relevant corpus (contrastive domain). Filtering ensures that those terms shall stay, which are more relevant to the target domain.

Domain relevance is used to measure the specificity of a term with respect to the target domain. It assigns scores to terms based on how relevant they are in the target domain and how irrelevant they are in contrastive domains. For this purpose, a list of contrastive domains as (D_1_,.., D_m_) is created. For the term t, domain relevance in target domain D_k_ is measured as (55)

(3) \begin{equation*} {DR}_{\left(t,k\right)}=\frac{P\left(t|{D}_k\right)}{\sum_{i=1}^mP\left(t|{D}_i\right)} \end{equation*}

where P(t|D_k_) and P(t|D_i_) are the probabilities of finding term t in the target domain D_k_ and contrastive domain D_i_, respectively. This probability can be estimated in terms of frequencies as

(4) \begin{equation*} Est\left({P}_t(d)\right)=\frac{f\left(t,k\right)}{\sum_{t\prime \epsilon{D}_k}f\left({t}^{\prime },k\right)}. \end{equation*}

On the other hand, Domain Consensus is used to find the terms that appear in several documents of target domain D_k_. It can be calculated as

(5) \begin{align*} {DC}_{\left(t,k\right)} ={\sum}_{d\ \epsilon \textit{D}_{k}} \Big({P}_{t(t)}.\mathit{\log}\frac{1}{P_{t(d)}} \end{align*}

where P_t_(d) stands for the probability of term t in document d of target domain D_k_.

The two measures are then integrated together using linear combination formula that is stated as

(6) \begin{equation*} {FinalScore}_{\left(t,k\right)}=\alpha{DR}_{\left(t,k\right)}+\left(1-\alpha \right){DC}_{\left(t,k\right)} \end{equation*}

where α is an experimental parameter that can vary from 0 to 1. A threshold is set and those terms are kept that are above thresholded score.

Guo *et al.* (56) utilized domain relevance and domain consensus measures for term extraction. Their algorithm obtained precision of 70% on Chinese text.

##### Co-occurrence analysis

Co-occurrence analysis is a concept extraction technique that locates the lexical units that occur together in pursuit of finding the implicit associations between various terms and concepts as well as extracting related terms. In documents, characteristic of co-occurrence appears in different forms such as phrase level co-occurrence of two words e.g. ‘real time’, ‘ballpoint’, and co-occurrence via common associations such as ‘Steve’ and ‘Apple’. Various co-occurrence measures are used to determine the associations and relations between terms such as Mutual Information (57), Chi-Square (58), Cosine Similarity, Dice Similarity (59), Kull-back Leiber Divergence (60) etc. Suresu and Elamparithi (61) employed co-occurrence analysis to extract domain-related terms for the extraction of concepts. Frikh *et al.* (62) also used this technique to extract cancer concepts. They used cancer related data set containing 52 758 documents, indexed from 26 different websites of cancer domain. Using Chi-Square approach, they obtained 67.35% precision and 59.93% recall.

##### LSA

LSA is an algorithm that is used in ontology learning for concept extraction. It is based on the idea that terms occurring together will be close in meaning. LSA applies the mathematical technique of singular value decomposition on term document matrix to reduce the dimension of data while maintaining the similarity structure. On the remaining terms, similarity measure (e.g. cosine similarity) is applied to find words that are similar to each other. Landauer *et al.* (63) and Lani *et al.* (64) used latent semantic analysis to find inherent relations by applying correlation techniques on this dimensionally reduced matrix which eventually yielded to concept formation.

##### Clustering for term/concept extraction

Clustering is an unsupervised learning approach in which objects are grouped into a number of clusters in such a way that objects within a group are more similar than the objects in other groups (65). K-Means clustering is an approach that clusters similar terms in the form of concepts. However, Karoui *et al.* (66) proposed an unsupervised hierarchical clustering approach named as Contextual Concept Discovery (CCD) for ontological concept extraction and compared it with K-means. For the evaluation of their proposed algorithm, they used HTML documents related to the tourism domain. They classified obtained clusters from these algorithms into three classes: advisable (validated by domain experts), improper (contains more than one concept) and unknown (neither validated by domain experts and does not contain any semantic relation). Their proposed CCD approach reduced the number of improper and unknown clusters obtained by K-means from 26.28 and 20.51 to 16.66 and 14.81%, respectively. Moreover, they obtained a greater number of advisable clusters, i.e. 68.52% as compared to 53.2% which were obtained from K-means clustering approach.

#### Relation Extraction

Statistical techniques are also used to extract taxonomic and non-taxonomic relations from the corpus. For taxonomic hierarchy induction, term subsumption and clustering techniques are used. On the other hand, ARM is used for non-taxonomic relations extraction.

**Term subsumption** Term subsumption finds hierarchical relations between terms by using the conditional probability of those terms in underlying documents. It looks for terms that are more general in the corpus. This algorithm states that term t is more general than term x if P(t|x) (probability of term t conditioned on the presence of term x) is higher than P(x|t) (probability of term × conditioned on term t) i.e. P(t|x) > P(x|t) where P(t|x) and P(x|t) are estimated as

(7) \begin{equation*} P\left(t|x\right)=\frac{No.\ of\ documents\ that\ contain\ term\ t\ and\ x}{No.\ of\ documents\ that\ contain\ term\ x}. \end{equation*}

(8) \begin{equation*} P\left(x|t\right)=\frac{No.\ of\ documents\ that\ contain\ term\ t\ and\ x}{No.\ of\ documents\ that\ contain\ term\ t}. \end{equation*}

The above equations refer that if term x is occurring in the documents that are a subset of the documents that contain term t, then t is more general as compared to x. In domain ontology learning, Njike-Fotzo and Gallinari (67) employed term subsumption technique to automatically extract generalization/specialization relationships between concepts extracted from LookSmart and NewScientist corpora. They compared the generated hierarchies with gold standard hierarchies and claimed that the performance of term subsumption is pretty good as it generated almost same hierarchies.

##### FCA

FCA is an interesting approach to build concept hierarchies in ontology learning. It relies on the basic idea that objects are connected with their characteristics (attributes). It takes an object attribute matrix as input and finds all natural clusters of attributes and objects together. It yields a lattice that has concepts and attributes in form of a hierarchy. Drymonas *et al.* (72) used FCA for taxonomic relationship extraction. They also compared it with agglomerative clustering based taxonomic relation extraction approach. On medical corpus, FCA obtained 47% precision, whereas agglomerative clustering managed to mark the precision of 71%. Their experiments showed that not only FCA was time complex [i.e. time complexity of O(2^n^)] but its results were also less accurate as compared to agglomerative clustering.

##### Hierarchical clustering

In the domain of ontology learning, hierarchical clustering is mostly used to find the taxonomic relations among data elements. It employs similarity measures (such as Cosine Similarity, Jaccard Similarity) to group the terms into clusters for the discovery of concepts and construction of hierarchy.

There are two strategies that are used to build hierarchy of clusters: (i) agglomerative clustering (bottom-up approach) (68) and (ii) divisive clustering (top-down approach) (69).

- Agglomerative clustering is a bottom-up approach. It considers every element as an individual cluster and combines the most similar elements into one cluster. The similarity between elements can be found using Cosine (59) or Jaccard Similarity measures. This method keeps on merging most similar clusters together until all elements are grouped into one universal cluster. Similarity between clusters is found using following three approaches: (i) single linkage, (ii) complete linkage and (iii) average linkage. Single linkage finds the two closest elements of both clusters and considers their similarity as cluster similarity. On the other hand, complete linkage uses the similarity of most dissimilar elements. Average linkage considers the average of both clusters similarities as cluster similarity. Agglomerative clustering gives rise to a hierarchy where elements are gathered as concepts in form of clusters. Certain thresholding criterion can be applied to stop the hierarchical clustering when most optimal concept clusters have been formed (68).
- Divisive clustering is a top-bottom approach. It considers all elements as one universal cluster and iteratively divides it into smaller clusters to form a hierarchy. The task of splitting a large cluster into small clusters can be performed using any clustering technique. K-means is another algorithm that is used to find concepts by forming clusters of terms (70).

Faure and Nédellec (71) used the bottom-up clustering technique to form concepts. They clustered the similar terms based on the similarity of their context in which these terms were used. At each step, they clustered two similar terms. Their approach was not completely unsupervised as a user was validating formed clusters at each stage. For the experimentation, they used cooking recipe data set that contained 1500 recipes. Using 50% data set they were able to get 92.1% accuracy and with 90% training data set they achieved 99.53% accuracy. The quality of their work was very high as most of the clusters they obtained were relevant.

In 2010, Drymonas *et al.* (72) presented a system for acquisition of ontologies from unstructured text. To extract taxonomic relations, they employed agglomerative hierarchical clustering and FCA on computer science and medical corpora (OHSUMED). The precision of agglomerative clustering on both computer and medical corpora was 71% whereas FCA yielded 44 and 47% precision, respectively. This revealed that clustering performed better than FCA for both corpora.

Caraballo (73) used agglomerative clustering approach to acquire hierarchy of terms in the form of hypernym–hyponym relationship. She collected data from the *Wall Street Journal* corpus that contained 50 000 distinct nouns. List of extracted hypernym–hyponym relations was given to different users for validation. For the best hypernym, 33% of her results were verified and for any randomly chosen hypernym 47.5% results were verified by all judges. In 2004, Maedche and Staab (60) presented an overview of clustering methods used for the construction of ontologies. They obtained ontology from different sources using hierarchical clustering methods. After experimentation, they claimed that divisive clustering is pretty complex that is why it is not frequently used for taxonomy induction (74).

Cimiano and Staab (75) proposed an oracle guided innovative hierarchical agglomerative clustering algorithm to learn concept hierarchies. This approach utilized hypernyms obtained from WordNet to guide the clustering process. For a given pair of terms, if they behave similarly in a corpus i.e. one is hypernym of other, then they were tagged as parent–child. But if both terms had same hypernym, they were added as siblings under the label of hypernym. Cimiano and Stabb (75) compared their approach with agglomerative clustering introduced by Caraballo (73). They claimed that their introduced clustering technique out performed agglomerative clustering with best F-measure of 21.4% in tourism domain. However, in finance domain, agglomerative clustering performed better with the F-measure of 18.51%.

##### ARM

ARM is a data mining approach that is used to discover hidden relations, associations and patterns among different elements in a database. In the domain of ontology, ARM is mostly used for non-taxonomic relation extraction. This idea came from market basket analysis in which seller happened to be curious to learn about what customers mostly buy and what types of products are bought together. To answer these question, it is important to learn the association between items I in database D, where I is the set of items say I = i_1_, i_2_, …, i_n_ and D = t_1_, t_2_ …, t_m_ is a set of transactions, respectively.

In ARM, rules are found that predict the co-occurrence of elements or items in databases. A rule is an implication of two-item sets such as X → Y where X and Y are non-empty subsets of I (set of items) such that X ∩ Y must be an empty set. For ARM, the following algorithms are commonly used:

1 Apriori algorithm
2 Frequent pattern (FP) growth algorithm
   * Apriori algorithm: In 1994, Agrawal and Srikant proposed this algorithm. It is used for mining the item sets that occur frequently in a data set and discovering associations between the elements of those frequent item sets. This algorithm is based on two steps:
   1. Generation of frequent item sets having support > minsup, where support of an item set is the fraction of transactions which contain that item set, and minsup is a threshold value that is used to filter least occurring item sets.
  (9) \begin{equation*} s= XUY/\left( no. of\ total\ transactions\right) \end{equation*}
   2. Generation of association rules (ARs) from those frequent item sets and prune them on the basis of confidence measure. Confidence c of a rule X → Y determines how often items in item set Y appear in transactions that contain X. Mathematically, it can be written as

(10) \begin{equation*} c=(XUY)/X. \end{equation*}

To perform the first step, frequent individual items or elements are collected from database and then they are extended by adding one element at a time until no further frequent items sets are found. For the second step, all possible combinations of items in a frequent item set mare formed and pruned on the basis of confidence measure value. Those rules are kept that fulfill a minimum confidence criterion.

In the domain of ontology learning, various term selection techniques are employed to extract terms. By considering these extracted terms as items, relationships using ARM can be found.

FP growth algorithm: In 2000, Han *et al.*, (155) presented FP growth algorithm that scanned the database two times in order to find the frequent pattern. It was developed to avoid the multiple scanning of the database for candidate set generation as it is pretty much time and resource consuming task. In this method, a compact tree-like structure is built to store information of frequent patterns occurring in the database. From these patterns, relations are extracted in the same ways as the Apriori algorithm extracts.

In the first scan, FP growth algorithm calculates the frequency count of each individual item in the database and places them in a frequency count table. In the second scan, each transaction of the database is sorted in descending order according to the frequency of its elements found in frequency count table. By using these sorted transactions one by one, a tree-like structure is built where each node holds an element and its frequency information. This is called Frequent Pattern Tree.

For the generation of rules out of frequent item sets, same confidence-based approach defined above is used for Apriori algorithm.

In 2016, Idoudi *et al.* (77) used ARM for ontology enrichment. They used Apriori algorithm to generate rules. For evaluation of generated rules, Liu *et al.*’s (76) operators were used, which classified these rules into three categories: known (already present in knowledge bases), unexpected (extracted rules that are new but not validated) and novel rules (extracted rules that are new and validated). For experimentation, they collected data of 1000 patient records from the hospital, Charles Nicolle in Tunisia and learned 1500 rules out of it. In their experiments, 68% rules were categorized as known, 31% rules were novel and 1% rules were unexpected.

AR mining was used by Drymonas *et al.* (72) for non-taxonomic relation extraction task of ontology development. They used computer science corpus and OHSUMED data set for experimentation. Non-taxonomic relation extraction using ARM revealed a precision of 72.5 and 71.8%, respectively, which is reasonably good.

Paiva *et al.* (78) used FP growth algorithm of ARM to enrich ontologies by finding the frequent item sets and generating rules out of them. In this method, a tree-like compact structure is built to find frequent item sets.

Ghezaiel *et al.* (79) presented an ontology enrichment process based on following two steps: extraction of new concepts and development of relations or associations between them and finding the most suitable place for novel association rules in existing knowledge bases. Their work mainly focused on discovering new ways for the extraction of new terms and relations along with already existing concepts in ontology.

Paiva (80) discovered association relations between medical concepts extracted from the data set containing information about the treatment of breast cancer. Their work was focused on finding semantic relations among the concept pairs, which were associated with each other. Similarly, Maedche and Staab (12) mined associations and built ontologies using textual data, which exist in the form of documents or in other forms such as web usage, web user profiles and web structure. For relation extraction, they used generalized ARM approach for the extraction of non-taxonomic relations. For evaluation, they used 2234 HTML documents with 16 million words. They obtained 51 000 linguistically related pairs, which contained 284 concepts and 88 non-taxonomic relations.

Fatemi *et al.* (81) employed AR along with manually discovered concepts for the extraction of new concepts. Their work clearly depicted that association rules play a key role in order to derive interesting correlations and associations present in the data. They performed experiments on TRECVid 2005 video corpus containing 43 907 shots and 449 manually annotated concepts. They discovered 287 new concepts using AR mining.

d’Amato and Learning (82) suggested to extend ontologies using the knowledge extracted from textual data. They extracted knowledge by discovering hidden patterns or associations among the concepts using ARs and proposed new axioms related to those concepts. The main idea of their work was to transform generated patterns or associations into formal rules. After the formation of rules, they used operators in order to differentiate redundant rules from non-redundant ones.

### ILP

ILP is a discipline of machine learning that derives hypothesis based on background knowledge and a set of examples using logic programming. In the domain of ontology, ILP is used at the final stage of ontology layer cake where general axioms are acquired from schematic axioms (axioms with both positive and negative examples and background knowledge).

Lima *et al.* (83) employed ILP technique to populate ontology from the web. In their work, they utilized two sources of evidence: WordNet (semantic similarity measure) and domain-independent linguistic patterns. They used patterns for the identification of candidates for class instance. Both of these evidence resources have been combined as a background knowledge for automatic acquisition of rules on the basis of ILP. They extracted 2100 sentences using Bing Search Engine API and evaluated performance with or without WordNet. They obtained 96% and 98% best possible precisions with and without WordNet, respectively.

Fortuna *et al.* (84) presented an innovative approach namely onto term extraction for the acquisition of topic ontology from textual documents. Their methodology was successfully utilized by ILP to generate the ontology of topics. For the experimentation of their proposed approach, they used the papers as documents, which were indexed in the database of ILPnet2 publications.

Seneviratne and Ranasinghe (85) described the use of ILP as a learning approach for the acquisition of ontological relations in a multi-agent system. In this multi-agent system, one agent used ILP for rule learning process while another agent used these rules to identify new relations. For the evaluation of their proposed approach they used Wikipedia web pages related to birds.

Lisi *et al.* (86) used ILP approach for relational learning as a huge amount of conceptual knowledge has been made available in the form of ontologies, usually formalized by Descriptive Logics (DLs). In their work, they considered the problem of combining ontologies and relational data and proposed ingredients of ILP as a solution to it. Their proposed approach was based on the deductive and expressive power of the KR framework DL+ data log. It allowed the strong integration of DLs and disjunctive data log with negation. They claimed that their approach laid the foundation of an extension of Relational Learning known as Onto-Relational Learning for ontologies.

Lisi *et al.* (87) described a logic-based computational approach to induce fuzzy ontologies automatically using ILP. They illustrated the usefulness of their approach by employing the proposed method on tourism domain. Their approach was a good contribution toward the management of automated fuzzy ontologies evolution.

To layout clear picture of all state of the art ontology learning techniques falling under different class (linguistic, statistical, and logical), we summarized their performance in various domains along with tools which have been used for their experimentation in Table 1.

**Table 1:** Performance Summary of Ontology Learning Techniques

**Techniques**		**Domain**	**Performance**	**References**	
				**Paper**	**Tools**
Linguistic Techniques					
Preprocessing	Berkley Parser	Tourism, Sport	Precision=95.7%	(28)	Text2Onto(75, 120, 121, 122) (http://neon-toolkit.org/wiki/1.x/Text2Onto.html), CRCTOL (28), https://nlp.stanford.edu/software/lex-parser.shtml, http://nlp.cs.berkeley.edu/
	Stanford Parser		Precision=90.3%		
	Syntactic Analysis for headword modifier	Chinese Text	Accuracy=83.3%	(29)	https://github.com/kimduho/nlp/wiki/Head-modifier-principle-(or-relation)
Relation Extraction	Lexico-syntactic Parsing	News	Accuracy=75.5%	(40)	Text2Onto (75, 120, 121, 122) (http://neon-toolkit.org/wiki/1.x/Text2Onto.html), CRCTOL (28), ASIUM (117, 118, 119) (http://www-ai.ijs.si/∼ilpnet2/systems/asium.html), TextStorm/Clouds (27, 123)
	Dependency Analysis	Bioinformatics	Accuracy=83.3%	(38)	
Statistical Techniques					
Term Extraction	C/NC Value	Medical	Precision=89.7%	(26)	OntoGain (72), https://github.com/Neuw84/CValue-TermExtraction
			Computer Science	Precision=86.67%	
	Contrastive Analysis	Chinese Text	Precision=70%	(56)	OntoLearn (49, 124, 55, 125), CRCTOL (28), OntoGain (72)
	Co-occurrence Analysis	Biomedical (Cancer)	Precision=67.3%	(62)	Text2Onto (75, 120, 121, 122) (http://neon-toolkit.org/wiki/1.x/Text2Onto.html), https://github.com/gsi-upm/sematch
	Clustering	Tourism	Accuracy=68.52%	(66)	ASIUM (117, 118, 119) (http://www-ai.ijs.si/∼ilpnet2/systems/asium.html), Text2Onto (75, 120, 121, 122) (http://neon-toolkit.org/wiki/1.x/Text2Onto.html), https://pythonprogramminglanguage.com/kmeans-text-clustering/
		Tourism	Accuracy=53.2%		
Relation Extraction	Formal Concept Analysis	Medical	Precision=47%	(72)	OntoGain (72), https://github.com/xflr6/concepts
		Computer Science	Precision=44%		
	Hierarchical Clustering	Medical	Precision=71%	(72)	Text2Onto (75, 120, 121, 122) (http://neon-toolkit.org/wiki/1.x/Text2Onto.html), https://github.com/mstrosaker/hclust
		Cooking	Precision=92.1%	(71)	
		Finance	F1 Score=18.51%	(75)	
		Tourism	F1 Score=21.4%	(75)	
	Association Rule Mining	Medical	Accuracy=72.5%	(72)	Text2Onto (75, 120, 121, 122) (http://neon-toolkit.org/wiki/1.x/Text2Onto.html)
Logical
	Inductive Logical Programming	English	Accuracy=96%	(83)	TextStorm/Clouds (27, 123) , Syndikate (126, 11), http://pyke.sourceforge.net/

In addition, we also cite the tools (column: Tools) and reference papers (column: Paper) against each performance benchmark produced by specific underlying ontology learning technique in different domains. Table 1 can prove a milestone for researchers and practitioners as it marks seven most prominent and widely used ontology learning tools with their respective methodology. Among all, Text2Onto, ASIUM and CRCTOL are considered hybrid ontology learning tools as they exploit both linguistic and statistical techniques in order to extract terms and relations from underlying corpus. Whereas OntoGain and OntoLearn solely utilize statistical-based methods in order to perform any ontology learning task. Similarly, TextStorm/Clouds and Syndikate use only logical techniques to acquire concepts and relations.

## Evaluation of ontology learning techniques

Assessing the quality of ontology acquisition is a very important aspect of smart web technology as it allows the researchers and practitioners to assess the correctness at lexical level, coverage at concept level, wellness at taxonomic level and adequacy at non-taxonomic level of yielded ontologies. Evaluation of ontology acquisition makes it possible to refine and remodel the entire ontology learning process in case of unexpected resultant ontologies, which do not fit with the specific requirements of a user. As discussed earlier, ontology learning is a multi-level process so this makes the evaluation process of ontology extraction pretty hard. Considering the complexity of evaluating domain ontologies, countless evaluation techniques have been proposed in the past couple of years and this area is still under continuous development. All proposed techniques fall under one of these categories, which are generally classified on the basis of kind of target ontologies and purpose of evaluation.

- Golden standard-based evaluation
- Application-based evaluation
- Data-driven evaluation
- Human evaluation

Table 2 gives an overview of ontology evaluation approaches against various supported evaluation levels of ontology learning.

**Table 2:** Overview of ontology evaluation approaches

Level	Golden standard	Application-based	Data-driven	Assessment by humans
Lexical, vocabulary, concept and data	x	x	x	x
Hierarchy and taxonomy	x	x	x	x
Other semantic relations	x	x	x	x
Context and application		x		x
Syntactic	x			x
Structure, architecture and design				x

This section highlights the research work done by many researchers and practitioners utilizing one of the mentioned evaluation techniques along with advantages, challenges and drawbacks.

### Golden standard-based evaluation

Golden standard-based evaluation is all about evaluating resultant ontology with a predefined benchmark or standard ontology. As gold standard ontology depicts an ideal ontology of a particular domain, assessing and comparing the learned ontology through this reference ontology can efficiently validate domain coverage and consistency. Golden standard can be a stand-alone ontology, statistical figures fetched from corpus or formalized by domain experts. Golden standard-based techniques are also known as ontology mapping or ontology alignment. All measures that come under the category of golden standard-based evaluation enable frequent and large-scale evaluations at multi-level. However, having an appropriate gold ontology may prove a huge challenge, since it needs to be the one that has been created with similar conditions and goals as suggested by the learned ontology. This leads to select either human-created taxonomies or reliable taxonomies of a similar domain as gold standard by most of the approaches. It is important to mention that all gold standard techniques mostly cover completeness, conciseness and accuracy factors for evaluation of learned ontologies.

Maedche and Staab (60) propose a set of similarity measures for ontology and empirical evaluation for different phases of ontology learning. They take ontologies as two-layer architecture comprising of lexical and conceptual layer. Considering this ontology model, they compute similarity between learned ontology and reference ontology, which is prepared by experts in tourism domain. They measure the similarity of ontologies on the basis of lexicon, semantic cotopy and reference functions. Moreover, Ponzetto and Strube (88) extracted a taxonomy from Wikipedia and compared it with a couple of gold standard taxonomies. At first, this technique utilizes a denotational mapper known as ‘lexeme-to-concept’ to map the extracted ontology. Finally, **semantic similarity is computed through WordNet using various measures: Leacock and Chodorow (****89****), Zavitsanos *et al.* (****90****), Trokanas *et al.* (****91****) and Sfar *et al.* (****92****) assess the learned ontology by comparing it with a gold standard ontology**. The proposed approach computes the similarity of two ontologies at lexical and relational level by transforming the ontological concepts and their attributes into vector representation. Likewise, Kashyap *et al.* (93) also exploited the similar approach by considering MEDLINE as corpus and MeSH thesaurus as benchmark to assess their extracted taxonomy. The assessment process actually compares the constructed taxonomy with the benchmark taxonomy using the following couple of metrics:

1. Content quality: It computes the extent of overlap among the labels of both taxonomies for sake of measuring precision and recall.
2. Structural quality: It computes the structural validity of all labels. For instance, if two labels are appearing in an ancestor–descendant relationship in first taxonomy then they must possess the same parent child relationship in other taxonomy.

Treeratpituk *et al.* (94) constructed a taxonomy from a corpus of larger text. They compared the constructed taxonomy with the six benchmark taxonomies. These taxonomies are topic specific and extracted from Wikipedia by exploiting their suggested GraBTax algorithm.

### Application-based evaluation

Application-based evaluation also referred as ‘Task Based Evaluation’ is an application and task-oriented evaluation as it evaluates given ontology by exploiting it in a specific application to perform some task. The outcome of particular task determines the goodness of specified ontology regardless of its structural properties. Task-based methodologies enable the detection of inconsistent concepts and allow to evaluate the adaptability of particular ontology by analyzing the performance of the specified ontology in the context of various tasks (95). In addition, task-based approaches are mostly getting exploited in the process of evaluating compatibility among employed tool and the ontology and measuring the required pace to complete the particular task. Application-based evaluation evaluates the correctness, coverage, adequacy and wellness of ontology in reference to other applications. For instance, an ontology is crafted in quest of improving the results of document retrieval. One may accumulate some sample queries to check if application retrieved more relevant documents after utilizing crafted ontology. In addition, it is important to mention that task-based evaluation measures mainly depend on the kind of task. In the case of document retrieval, traditional measures of information retrieval such as F-score can be used (96, 97). Lozano-Tello *et al.* (98) proposed a technique that enables the users to determine the suitability and appropriateness of existing ontologies with the requirements of their respective systems. Porzel and Malaka *et al.* (99) evaluated the exploitation of ontological relations in speech recognition. Human-generated gold standard is used to compare the outcome of the speech recognition system. It is important to mention that application-based evaluation has several shortcomings, which are highlighted as below:

- Ontology gets evaluated after getting exploited in a particular way by a specific application for a particular task; therefore, it is pretty hard to generalize its performance.
- Ontology can be a minor component of an application so its impact over the results may be indirect and small.
- Various ontologies can be compared if they all can be embedded into the same application for the same task.

Moreover, Haase and Sure (100) assess the quality of specific ontology by finding the extent to which it enables the users to acquire relevant individuals in particular search. They introduce a cost intensive model to figure out the required user’s effort against desired relevant information. This cost is computed through the complexity of constructed hierarchy in form of breadth and depth.

### Data-driven evaluation

Data-driven or so-called Corpus-based evaluation (96) utilizes existing domain-specific knowledge sources (usually textual corpora) to assess the extent of coverage by specific ontology in particular domain. The major advantage of this approach is enabling the comparison of one or more target ontologies with a specific corpus. Like golden standard-based approach, it also covers the similar evaluation criteria comprising of completeness, conciseness and accuracy of learned ontologies. The major challenge of data-driven approaches is to find a domain-specific corpus that is much easier than finding a fine domain-specific benchmark ontology. For instance, Jones and Alani (101) utilized Google as the search engine in order to find a corpus against a specific user query. After expanding the user query by exploiting WordNet, the top 100 pages of Google results are taken as the corpus for the sake of evaluation. Many researchers performed the corpus based evaluation. For example, Brewster *et al.* (102) explained the number of techniques and methodologies for assessing the structural fit among ontology and particular domain knowledge, which exists like text corpora. They acquire domain-specific terms from textual corpora by utilizing latent semantic analysis. The extent of overlap among domain-specific terms and terms revealing in a particular ontology (i.e. concepts names) are used to compute the fit among the ontology and corpus. Moreover, they proposed a probabilistic methodology to determine the best ontology among all candidate ontologies. Sordo *et al.* (39) used it to evaluate the music relations extracted from unstructured text. Likewise, Patel *et al.* (103) assessed the coverage of specific ontology by retrieving textual data such as concepts names and relations from it. The acquired textual data is exploited as a source of input to a fine text classification model, which is trained by utilizing various standard machine learning methodologies.

### Human evaluation

Human evaluation of ontologies is generally based on defining and formulating various decision criteria for the selection of best ontology from a specified set of candidate ontologies. A numerical score is assigned after evaluating ontology against each criterion. Finally, a weighted sum is calculated through criterion scores. This kind of evaluation is also called ‘Criteria Based Evaluation’ (96). Criteria-based evaluation is extensively getting used in many contexts for the selection of best ontology (i.e. grant applications, tenders etc.). The major shortcoming of criteria-based evaluation is the requirement of high manual cost in terms of time and effort. However, this approach is deprecated and not used very often nowadays. Researchers did quite some work over this approach. For example, Burton-Jones *et al.* (104) proposed a list of 10 criteria comprising of richness (number of syntactic features present in formal language are utilized by specific ontology), lawfulness (syntactical errors frequency), interpretability (determining the existence of ontology terms in WordNet), clarity (number of terms senses present in WordNet), consistency (number of inconsistent concepts), accuracy (number of false statements in the target ontology), comprehensiveness (total concepts in the target ontology, compare to the average for the entire repository of ontologies), authority (number of ontologies utilizing the concepts from target ontology), history (number of accesses have been made to target ontology in comparison of other candidate ontologies) and relevance (total statements which involve significant syntactic features). Similarly, Fox *et al.* (105) present a set of criteria that is more inclined toward manual evaluation and assessment of ontologies. Lozano-Tello *et al.* (106) formulate a set comprising of 117 criteria, grouped in a framework of three levels. They assess taxonomies on the basis of multi-level properties comprising of cost, design qualities, language properties and tools through the assignment of some scores. Moreover, criteria-based evaluation can also be classified in two categories which are discussed below.

- Structure-based evaluation
  Structure-based methodologies explore and measure different structural properties in quest of evaluating specified taxonomy. Most proposed structure-based techniques fully automate the entire evaluation process. For example, one may compute the relational density of all existing nodes and an average of taxonomic depth. Like, Fernández *et al.* (107) examine the effect of various structural ontology methodologies in context of ontology quality. After extensive experimentation, they conclude that lavishly populated ontologies in terms of high depth and breadth values have more chances of being correct. Besides, Gangemi *et al.* (108) assess ontologies on the basis of presence of cycles in a directed graph.
- Complex- and Expert-based evaluation
  Complex- and expert-based evaluation measures are in high numbers, which try to embed various aspects and properties of ontology quality. For instance, Alani and Brewster *et al.* (109) add many ontology evaluation measures such as density, betweenness and class matching measures in ‘AKTiveRank’ system. Moreover, Guarino and Welty (110) assess ontologies through a system known as ‘OntoClean’. OntoClean is based on a set of notions comprising identity, essence and unity. They exploit the OntoClean notions to characterize and explore the suggested meaning of classes, relations and properties that actually prove significant to build up a specific ontology.

## Ontology learning data sets

This section summarizes the characteristics of commonly used data sets and systems in ontology learning. For the development of ontologies using ontology learning techniques, data sets containing unstructured domain-specific documents are used. For the biological domain, most of the researchers use OHSUMED (http://davis.wpi.edu/xmdv/datasets/ohsumed) (111, 112, 113) and Genia Corpus (http://www.geniaproject.org/genia-corpus) (114, 115) for experimentation. Similarly, in traveling and tourism domain, data sets for ontology learning are Mecklenburg Vorpommern (116, 75) and Lonely Planet (http://www.lonelyplanet.com/destinations) (116, 75). Two large data sets of news domain namely British National Corpus (http://www.natcorp.ox.ac.uk/) (97) and Reuters-21578 (https://archive.ics.uci.edu/ml/datasets/reuters-21578+text+categorization+collection) (113, 97) are also extensively used for experimentation and evaluation of different ontology learning systems. Table 3 illustrates the characteristics of six data sets.

**Table 3:** Summary of Popular Datasets

Corpus	No. of documents	Domain	Tokens
Mecklenburg Vorpommern	1047	Tourism	332000
Lonely Planet	1801	Traveling	1 Million
British National Corpus	4124	News	100 Million
Reuters-21578	21578	News	218 Million
OHSUMED	348566	Biological	NA
Genia Corpus	2000	Biological	400000
Planet Stories	307	Stories	NA

## Industrial applications of ontology learning

A large amount of unstructured and semistructured data is being generated every second in the world. If we talk about statistics of data generation, almost 2.5 quintillion bytes of data were generated every day in 2017, which is a humongous amount ( https://www.ibm.com/blogs/insights-on-business/consumer-products/2-5-quintillion-bytes-of-data-created-every-day-how-does-cpg-retail-manage-it/). These data are distributed over the internet at various websites in such a way that it is totally disconnected. Storing such gigantic amount of data requires a lot of resources. Moreover, it is extremely difficult to process such data is order to find useful information. This marks the desperate need of a knowledge representation model, which shall store such data in a more structured way to enable fast processing and quick retrieval at large scale. The model that enables structured representation of data is known as ontology.

Ontologies are being extensively used in information retrieval, question answering and decision support systems. This section illustrates applications of ontology in diverse industries such as oil and gas industry, military, e-government, e-health and e-culture etc.

### Oil and gas industry

Oil and gas industry is one of the most data intensive industry that is generating a huge amount of important data every day. Data are being generated from various sources in the form of oil wells data, seismic data, drilling and transportation data, customer data and marketing data. Since it is one of the industry that controls the balance of power in the world, these data along with its semantic are of significant importance as it can be used to derive very useful information. Soma *et al.* (127) presented a reservoir management system that uses the semantic web to access and enhance the view of information present in its core knowledge base. Fluor Corporation’s Accelerating Deployment of ISO 15926 (ADI) (150, 151) project converts ISO 159263 Part 4 (a resource of oil and gas industry that has descriptions of plant objects) into RDF/OWL form to make it process-able by computer systems. Norwegian Daily Production Report project implemented ontology based on ISO 15926 standard to make data comparison and retrieval easy. Moreover, workflow and quality of oil and gas industry can be further improved by utilizing the semantic web concepts by integrating the semantic web with Internet of Things.

### Military technology

Diverse military technologies such as drones and weaponized mobile robots are producing exponentially large battlefield information. Technologists are using the semantic web to manage massive data load and assist decision analysis during the battle by utilizing the significant information produced by all auto-military units. In addition, ontologies are being constructed to conjure up battlefield information for quick retrieval. Halvorsen and Hansen (152) provided an integrated approach to access military information, which uses RDF representation and serialization mechanism between various systems and uses SPARQL as communication protocol. This approach can be used for threat detection by reasoning over the information provided in RDF triplets~(128).

In quest of standardizing available information, decision making and exchanging information effectively, technologists introduced diverse ontologies like MilInfo (129) and Air Tasking Order (ATO) (130). The ATO helps to assign the aircraft missions. Besides this, Tactic Technique and Procedure Ontology (131) as well as Battle Management Ontology (132) are some more ontologies to assist military decision making and shared information access. Another possible ontology could be the soldier ontology (http://rdf.muninn-project.org/ontologies/military.html), which can be generated by making use of the data of both on duty and retired soldiers. This type of ontology can help in selection of soldiers for specific missions and keeping tracks of retired senior soldiers.

### E-government

Incorporation of ontology and the semantic web in e-government portals can be very fruitful. Instead of relying only on text, the underlying ontology can be used to extract the information that is semantically more meaningful to the query. Such portals are more efficient than simple traditional search portals, which do not consider semantics. Various governmental departments will be able to keep their knowledge bases in sync by using the underlying ontologies.

Rui *et al.* (133) presented the concept of semantic information portal that utilized semantic search algorithm. They not only proposed but also implemented the algorithm to retrieve semantically correct results against queries. On the other hand, Haav (134) described a process with which ontologies can be created for e-governmental data. By making use of these ontologies and semantics, government can manage their resources effectively and improve the planning and development policies.

### E-business and E-commerce

E-business and e-commerce have also started utilizing the powers of the semantic web to make important business decisions and to develop smart systems for end users by handling massive available data efficiently using ontologies. GoodRelations is one such ontology introduced by Hepp (135). The ontology is essential for any semantic based web platform as it models various e-commerce concepts like products, prices, discount offers, sales offers etc. LIB2CO created by Akanbi (136) is another integrated semantic web platform that offers two major agents. One is search agent that retrieves semantically correct results to consumer queries by analyzing the metadata attached to products. The other is ontology agent whose task is to organize all the products into an ontology so that the search agent can find it effectively.

Ontologies are also helpful in commerce matchmaking where the best compatible services and goods are selected for the user. Paoloucci *et al.* (137) developed such a system which comprises of various ontologies and a matchmaker. Besides this, a security ontology developed by Ekelhart *et al.* (138) played its part in the security infrastructure of ontology based ecommerce and e-business.

### E-health and life sciences

E-health and life sciences industry are also in quest of feeding patient data electronically for better processing and quick retrieval. In order to make this data useful for artificial intelligence applications, semantics behind the data need to be involved to enable automatic decision making.

European Patient Summary (153) is one such project whose backbone lies in the semantic web technologies. Besides this, ontologies and semantics have also been used by Podgorelec and Pavlic (139) to store and integrate the data about Mitral Valve Proplapse syndrome. Kim and Choi (140) presented an electrocardiography ontology for heart diseases and used it to create a knowledge base. Ganguly *et al.* (141) also worked on eHealth-based ontologies by addressing the issue of mismatch between conceptual hierarchies in ontologies. Some other applications of ontology learning for eHealth are present in the form of ontologies like Human Phenotype Ontology (142), Translational Medicine Ontology (143) and SNOMED CT (Systemized Nomenclature of Medicine Clinical Terms) (144).

### Multimedia and E-culture

Annually, a huge amount of multimedia content is released on the internet, which includes >2500 movies and 1 million songs. The metadata attached to these multimedia contents along with its semantics can prove to be very helpful for multimedia companies as they can use it to build precise and accurate recommendation systems for their customers.

Retrieving relevant images, video contents and songs is one of the tasks that can be done using ontologies and semantics. Fan and Li (145) used an ontology-based reasoning system to retrieve the images relevant to the queries. Besides this, an animal ontology has been used in animal domain by Wang *et al.* (146) to retrieve and annotate animal images. Liu *et al.* (147) used reverse engineering process and generated an image ontology from images data. Ontologies have found their application in video annotation and retrieval process by utilizing the semantics of events happening in the video. Ballan *et al.* (148) presented one such framework for annotation and retrieval of video content.

### Investigative and digital journalism

The semantic web and usage of numerous ontologies have taken journalism to next level by enabling the exploration of hidden and non-achievable information for all journalist through deeper search. For instance, Panama Papers is a gigantic list of documents that contains information about organizations and individuals who dodge sanction and taxes. Unfortunately, its information was non-accessible to journalists. Ontotext (https://ontotext.com/) company constructed an ontology from the list of these documents to give them more structure and meaning. It also enabled querying mechanism using SPARQL. Similarly, Trump World Data is another result of investigative journalism which has been transformed into structured text for easy information access.

## Future directions

Ontology learning is a multidisciplinary task that extracts important terms, concepts, attributes and relations from unstructured text by borrowing techniques from different domains like text classification, natural language processing machine learning etc. These domains are research extensive and still developing. Natural language processing has various bottlenecks such as part of speech tagging, relation extraction from unstructured text, co-reference resolution and named entity recognition. From results discussed in the section entitled Linguistics for pre-processing, it can be concluded that techniques like PoS tagging and parsing can lead toward the development of better ontologies. With the advancement in NLP techniques, improved PoS taggers and parsers are being introduced that needs to be merged into ontology learning systems for better performance. In text classification, researchers are developing new algorithms to select highly discriminative features among the classes. There are many term selection algorithms available in these domains that [Bi-Normal Separation, Normalized Difference Measure, Odds Ratio, Poisson Ratio Balanced Accuracy Measure (ACC2) and Distinguishing Feature Selection (154)] needs to be introduced in ontology learning for the extraction of terms and concepts.

As far as machine learning is concerned, ontology learning borrows various techniques from this domain such as clustering and ARM. However, improvements can be made by incorporating the domain of deep learning into these algorithms. Besides this, the exponential growth of textual data on the web is heavily influencing various methods used at different levels of ontology learning. It can be said that the future of ontology learning will be led by the immense amount of unstructured web data. We propose following future directions to further improve ontology learning process:

1. Use of social media for data validation
2. Language independent ontology learning
3. Scalability of existing ontology learning techniques to cater larger data sets
4. Use of crowdsourcing and human-based computation games to perform ontology post processing
5. Development of more formal or heavyweight ontologies

This section summarizes five prominent challenges of ontology learning and discusses above mentioned future directions in context of these challenges.

Challenge 1: The immense amount of web data exists in different formats and languages. This leads to the production of conflicting and inconsistent ontologies.

Proposed solution:

To resolve this issue, we propose look for approaches to integrate and homogenize such data. This field has not yet gained enough attention by ontology learning community. We also propose use of cross language ontologies in quest of resolving such issues. There exists a need to develop advanced algorithms for ontology learning which are independent of language barriers. Since ontologies are actually shared conceptualization, they should be free of lexical information. For example, orange should not be portrayed lexically as ‘orange’ but rather as a form to which oranges of all languages can be mapped to.

Challenge 2: **Ontology learning is still a developing field where each task of ontology learning layer cake is vast research that needs improvement. Each stage is dependent on results of the previous stage. If one stage produces wrong information, it will affect the later stages and it would eventually produce low quality ontologies.** For example, if a faulty relation <VladmirPutin> <is−a> <president of Italy> occurs frequently in data, ontology learning methods will extract it and add it to final ontology. This will contaminate underlying knowledge base.

Proposed solution:

To ensure data validity we propose use of social web and folksonomy (collaborative tagging). We can assess the validity of learned ontology by asking users of social media to tag extracted concepts and relations either as correct or incorrect. By comparing the total number of users tagging them correct and incorrect, we can develop some level of trust for our learned ontology.

Challenge 3: Scalability of ontology learning techniques to accommodate larger data sets is another major challenge. Most of the techniques and tools used in state-of-the-art ontology learning methodologies are designed for smaller data sets. Such techniques and tools, when applied on bigger data sets, tend to produce inefficient results.

Proposed solution:

We suggest an increase in research to scale the present techniques up to certain level to accommodate larger data sets without compromising on the efficiency and quality. This can be done by introducing some community challenges like BioASQ, BioCreative, TREC etc. Various incentives in these challenges will be attractive for researchers and improvements will be made to tackle this challenge.

Challenge 4: The quality of learned ontologies is affected by the human intervention. We can say that the quality of learned ontology is directly proportional to human intervention. This is why semi-automatic ontology acquisition process tends to produce good ontologies. For automatic ontology learning process, **a reasonable amount of post processing is required to boost the quality of ontology**, which is another massive drawback of fully automated ontology acquisition. It puts a lot of burden on knowledge engineers and domain experts.

Proposed solution:

This post processing stage somehow must be integrated with the original ontology learning framework. To reduce this overhead, we propose to utilize the extensive amount of research in the field of crowdsourcing and human-based computation game (games with purpose). These can help lower the cost of ontology revision by involving non-expert humans and interacting with them to achieve post processing goals.

Challenge 5: Lastly, we predict a need to shift from lightweight ontologies to more formal, heavyweight ontologies in the future.

Proposed solution:

To tackle this problem, there is a strong need to strengthen axiom learning techniques so that in future formal ontologies take the center stage.

Above aforementioned challenges and future direction are summarized in Table 4.

**Table 4:** Summary of Ontology Learning: Challenges and Future Directions

	Challenge	Proposed Solution
1	Diversity of formatted data, multi-lingual data	Novel approaches to integrate and harmonize data Cross-language ontologies advanced algorithms for ontology learning
2	Lack of automatic ontology validation, faulty ontologies	Use of social web, collaborative tagging and folksonomy Use of search engines for answer validation
3	Scalability of ontology learning techniques	Increase in research to accommodate larger datasets Arrangement of community challenges by governing bodies to increase the research scale of ontology learning techniques
4	Requirement of human intervention for better quality of learned ontologies	Need of automatic post processing techniques Integrate post processing framework with ontology learning framework to boost the quality of ontology Use of research in the fields of crowdsourcing and human-based computation games
5	Lack of heavy weight ontologies	Strengthen axiom learning algorithms

## Conclusion

This paper summarizes ontology learning techniques along with evaluation measures and highlights applications of ontology learning in various domains. We observed that a hybrid approach comprising of both linguistic and statistical techniques produces better ontologies. However, it is difficult to find the best technique among all as the performance of ontology learning techniques is highly dependent on efficient preprocessing of data in target domain. After critically analyzing the literature of ontology learning, following trends are observed: for term and concept extraction, many researchers prefer to use statistical techniques; however, for relation extraction, there is an inclination of use toward agglomerative clustering and ARM. We also overviewed various evaluation techniques for ontology learning and have found that the best form of evaluation is human-based evaluation. In addition, we also mark most widely used ontology learning tools along with their respective methodology and target domain. Applications of ontology learning in industries such as oil and gas, military and e-health etc. are also discussed. Lastly, we provide comprehensive information about ontology learning challenges. We also propose their solutions to further improve the process of ontology learning by showing directions for answer validation, language-independent ontology generation and crowdsourcing usage for automatic ontology post processing.
